# Genetic Map of Mango: A Tool for Mango Breeding

**DOI:** 10.3389/fpls.2017.00577

**Published:** 2017-04-20

**Authors:** David N. Kuhn, Ian S. E. Bally, Natalie L. Dillon, David Innes, Amy M. Groh, Jordon Rahaman, Ron Ophir, Yuval Cohen, Amir Sherman

**Affiliations:** ^1^Subtropical Horticulture Research Station, United States Department of Agriculture—Agriculture Research ServiceMiami, FL, USA; ^2^Department of Agriculture and Fisheries, Centre for Tropical Agriculture, Horticulture and Forestry ScienceBrisbane, QLD, Australia; ^3^International Center for Tropical Botany, Florida International UniversityMiami, FL, USA; ^4^Department of Fruit Tree Sciences, Plant Sciences Institute, Agriculture Research OrganizationRishon Letzion, Israel

**Keywords:** genetic recombination map, *Mangifera indica* L., SNP marker, trait association, polyembryony

## Abstract

Mango (*Mangifera indica*) is an economically and nutritionally important tropical/subtropical tree fruit crop. Most of the current commercial cultivars are selections rather than the products of breeding programs. To improve the efficiency of mango breeding, molecular markers have been used to create a consensus genetic map that identifies all 20 linkage groups in seven mapping populations. Polyembryony is an important mango trait, used for clonal propagation of cultivars and rootstocks. In polyembryonic mango cultivars, in addition to a zygotic embryo, several apomictic embryos develop from maternal tissue surrounding the fertilized egg cell. This trait has been associated with linkage group 8 in our consensus genetic map and has been validated in two of the seven mapping populations. In addition, we have observed a significant association between trait and single nucleotide polymorphism (SNP) markers for the vegetative trait of branch habit and the fruit traits of bloom, ground skin color, blush intensity, beak shape, and pulp color.

## Introduction

Mango (*Mangifera indica*) is one of the most important fruit crops of the world due to its large fruit with a soft, sweet pulp. A subtropical group in the Indian sub-continent is characterized by monoembryonic seed and a tropical group in the south-east-Asia region is characterized by polyembryonic seed (Mukherjee and Litz, 2009).

Mango has been widely cultivated in India and Southeast Asia for thousands of years. In the fifteenth and sixteenth centuries, Portuguese and Spanish traders spread mango to other tropical and subtropical regions of the world (Mukherjee and Litz, 2009). Early in the twentieth century, cultivars from the Indian and Asian regions were combined in a new center of mango development in Florida, where many cultivars were selected and disseminated. These cultivars, selected for milder taste and aroma, colorful skin, and larger fruit size, are still the major cultivars used today in international trade.

Mango is now grown throughout the sub-tropical and tropical world in 99 countries with a total fruit production of 34.3 million tons of fruit per annum (Galán Saúco, 2013). The majority (76%) of world production comes from Asia, with the Americas (12%), and Africa (11.8%) the second and third largest producers. India is the largest producer, growing over 18 million tons (MT) primarily for domestic consumption, followed by China (4.5 MT) Thailand (3.1 MT), Indonesia (2.6 MT), and Mexico (1.9 MT) (Galán Saúco, 2013). Although, Mexico is fifth in production it is first in export to the USA, which is 43% of the global import market.

Around the world there are hundreds and possibly thousands of different mango cultivars and selections, most of which are only grown and marketed locally. Relatively few cultivars are traded internationally due to the highly specific requirements for cultivars with favorable color, storage, and shipping traits.

Mango is suggested to have a partial allopolyploid genome based on cytogenetics (Mukherjee, 1950). However, genetic markers for mango have been reported to be inherited in a disomic fashion by several authors (Duval et al., 2005; Schnell et al., 2005, 2006; Viruel et al., 2005) suggesting that mango may be treated as diploid. Mango has a total of 40 chromosomes, which suggests a haploid number of chromosomes as 20 and similarly 20 linkage groups. The haploid genome size is estimated at ~439 Mb (Arumuganathan and Earle, 1991).

To date the development of genetic and genomic resources in mango have been limited and have not greatly contributed to mango breeding around the world. An early, very limited genetic map of mango produced by Kashkush et al. (2001) was not sufficiently resolved to be useful for marker assisted selection (MAS) or trait association to markers. Recently, a high resolution map of mango has been produced by Luo et al. (2016) that may prove more useful. Several transcriptomes from different mango tissues have been produced (Pandit et al., 2010; Azim et al., 2014; Luria et al., 2014; Wu et al., 2014; Dautt-Castro et al., 2015; Sherman et al., 2015). In 2016, Kuhn et al. (2016) identified ~400,000 single nucleotide polymorphism (SNP) markers using a reference transcriptome from “Tommy Atkins” and sequences of expressed mRNA from 17 genetically diverse cultivars. The genetic diversity of mango has been explored by different groups with a variety of markers, who all found a narrow genetic basis among the commercial cultivars grown and traded internationally (Schnell et al., 2006; Dillon et al., 2013; Sherman et al., 2015). An increase in the number of unbiased markers and a highly resolved genetic map are essential molecular tools for mango breeders if the power of genomics is to drive future progress of breeding for improved mango cultivars.

The current improved commercial cultivars have typically been selected from open pollinated seedling progeny and then vegetatively propagated to maintain genetic uniformity (Bally et al., 2009). The continual demand for new and improved cultivars with superior production and quality traits is a challenge for breeders relying on traditional breeding techniques. Factors that limit progress in traditional fruit tree breeding are the long juvenile phase, long generation time, and large resource requirements in field area and personnel for maintaining and evaluating hybrid populations. In addition to these restraints, mango breeders are faced with high heterozygosity, polyembryony, low crossing rates (0.1%) from high numbers of flowers per panicle, a very high level of fruitlet drop, and only a single seed per flower resulting in a low number of fruit (0.1% of flowers), all of which makes the task of active manual crosses challenging (Bally et al., 2009). There is also little knowledge of the heritability of most of the important horticultural traits in mango (Schnell et al., 2006). Finally, the lack of genotypic and phenotypic diversity among the current commercial cultivars may reduce breeding efficiency if used as parents in breeding programs. Adoption of molecular genomic tools has the potential to estimate genetic diversity of potential parents, identify markers associated with important horticultural traits and, in general, improve the efficiency of mango breeding programs.

Major mango breeding/selection programs exist in India, Australia, Brazil, and Israel, and although each program has breeding goals specific for their industries, they share many productivity and quality goals. Full-sib hybrid populations from two known parents with differing horticultural traits, such as hand pollinated populations, are more effective for breeding progress than half-sib populations from open pollinated maternal parents. Genetic maps that are based on segregating full-sib hybrid populations are a powerful tool to identify linkage between horticultural traits and molecular markers for MAS as seen in other tree fruit crops (Ogundiwin et al., 2009; Martínez-García et al., 2013; Harel-Beja et al., 2015).

Linking and mapping important mango traits with molecular markers will improve the efficiency of mango breeding. One of the traits of mango that is very distinct is polyembryony in which multiple apomictic embryos develop from the maternal nucellar tissues around the fertilized egg in addition to a single zygotic embryo (Asker and Jerling, 1992). Most mango cultivars originating in India are monoembryonic, while cultivars originating from Southeastern Asia are usually polyembryonic (Litz, 2009). Trees developing from the apomictic embryos of polyembryonic mangos are genetically similar to the maternal tree. This property provides an easy method of clonal propagation, which may be used commercially to produce uniform rootstocks in addition to allow commercial cultivars to grow on their own roots. Such clonal rootstock can be well-adapted to the local growing conditions and soils.

Although, polyembryony in mango was originally thought to be controlled by recessive genes (Sturrock, 1968), later genetic evidence suggested that polyembryony in mango is controlled by a single dominant locus (Aron et al., 1998). Polyembryony in citrus may be controlled by more than one gene as several sequences (Nakano et al., 2012) and genes associated with polyembryony have been identified (Nakano et al., 2013; Kumar et al., 2014).

In this study, we generated a mango consensus genetic map, a valuable tool that can be used to improve the efficiency and overcome the challenges facing mango breeding programs. We used the genetic map to identify markers and regions of the genome that are associated with important horticultural traits such as embryo type, branch habit, bloom, ground skin color, blush intensity, beak shape, and pulp color.

## Materials and methods

### Mapping populations

Seven mapping populations were used to make the consensus map (Table 1). The four mapping populations from Australia share a common paternal parent, Kensington Pride (KP). In addition, the cultivar NMBP1243, the maternal parent of one of the mapping populations, is a progeny of the Irwin (I) × KP population. The Brazilian population Haden (H) × Tommy Atkins (TA) share both parents with the self-pollinated populations of H and TA from the Subtropical Horticulture Research Station (SHRS). The TA self-pollinated population was generated by germinating and genotyping fruit from a commercial grove planted with only TA. The H self-pollinated population was generated by germinating and genotyping fruit from an isolated tree at SHRS.

**Table 1:** **Number of progeny and the sources of seven hybrid mapping populations used to create the consensus genetic map**.

**Population name**	**Number of individuals**	**Source of population**
Tommy Atkins × Tommy Atkins (TA × TA) (Self-pollinated)	60	USDA-ARS, SHRS, USA^a^
Tommy Atkins × Kensington Pride (TA × KP)	100	DAFQ, Australia^b^
Haden × Tommy Atkins (H × TA)	225	Embrapa, Brazil^c^
Haden × Haden (H × H) (Self-pollinated)	40	USDA-ARS, SHRS, USA^a^
Irwin × Kensington Pride (I × KP)	180	DAFQ, Australia^b^
NMBP1243 × Kensington Pride (NMBP1243 × KP)	100	DAFQ, Australia^b^
Creeper × Kensington Pride (Cr × KP)	70	DAFQ, Australia^b^

*Populations were named maternal parent × paternal parent*.

a *United States Department of Agriculture-Agricultural Research Service, Subtropical Horticulture Research Station, United States of America*.

b *Department of Agriculture and Fisheries, Queensland, Australia*.

c *Brazilian Agricultural Research Corporation (Embrapa), Pernambuco, Brazil*.

### SNP containing sequences

SNP containing sequences came from three different sources: Department of Agriculture and Fisheries, Queensland (DAFQ), Australia, SHRS, USA and the Agriculture Research Organization (ARO), Israel (Table 2). The SHRS SNP markers were identified as described in Kuhn et al. (2016). The ARO SNP markers were identified as described in Sherman et al. (2015). The DAFQ SNP markers were identified from sequence data described in Hoang et al. (2015).

**Table 2:** **Source of SNP assays used in the construction of the consensus genetic map for mango**.

**SNP assay**	**Number of SNPs**	**Contributors and citations**
Australia	144	DAFQ Hoang et al., 2015
Israel	384	ARO Sherman et al., 2015
US	526	USDA-ARS SHRS Kuhn et al., 2016
Total	1,054	–

### DNA isolation

DNA for genotyping was isolated from the leaves of individual progeny in the mapping populations as in Kuhn et al. (2016). Once isolated the DNA was quantified by fluorescence on a fluorescence plate reader (BioMark, Inc.) and normalized to 10 ng/uL on a liquid handling robot (Hamilton, Inc., Reno, NV, USA).

### SNP assays

All 1,054 SNP assays were produced from SNP containing sequences by Fluidigm (South San Francisco, CA, USA) and assayed on a Fluidigm EP-1 platform.

### Data reformatting

Perl scripts (available on request) were written to reformat data from all 1,054 markers generated by the Fluidigm EP-1 platform. Data from all mapping populations for all 1,054 markers were appended into a single file. Due to the large size of the combined data file, the initial analysis was performed on a 32 core Linux cluster followed by data reformatting and analyzing with scripts that produced csv files for export to Excel. Off type individuals, i.e., not hybrid progeny of the parents of the population, were identified by multiple occurrence of genotypes that could not have been inherited from the parents and were removed from the dataset. Markers with >5% missing data were also removed from the dataset. In the resulting edited dataset, individual progeny with >5% missing data were then removed. SNP markers that were homozygous for both parents in a population were removed because they would not be informative for finding recombination events. Selection was made for markers with disomic inheritance segregation ratios. SNP markers with segregation ratios differing by more than 20% from the expected disomic genotypic frequency or allelic frequency were removed from the dataset. Such markers had either aberrant segregation ratios based on the parental genotypes or segregation ratios indicative of tetraploid inheritance.

### Genetic mapping

Two mapping programs, JoinMap4 (Kyazma B.V.®, Wageningen, Netherlands) and OneMap (Margarido et al., 2007) were used to create genetic maps for each of the seven mapping populations (Table 1). Each program has advantages and they were used in conjunction as follows. OneMap was used to identify 20 groups because it could be run recursively to identify a predetermined number of groups. OneMap was run individually for all seven mapping populations with recursive runs that increased the acceptable likelihood of the odds (LOD) threshold (increasing by increments of 0.1) until 20 linkage groups (LGs) were achieved with a minimum of 10 markers per LG.

The TA × KP population analysis in OneMap produced a map with the most markers per LG (480 markers total were grouped with at least 20 per LG). These individual LGs were used to force the initial marker grouping in JoinMap4. All calculations in JoinMap4 were conducted with default parameter settings for the population, grouping, and Maximum Likelihood (ML) mapping. JoinMap4 has a function that allows ungrouped markers to be added to groups based on an association score, the Strongest Cross Link value (SCL value). Any marker with an SCL value ≥5.0 was added to its SCL group. This was repeated until no markers had SCL values >5.0. Loci that were marked as identical to another locus were also included in groups. Markers were removed from linkage groups if they prevented mapping in JoinMap4 or if they were >200 cM distance from the next closest marker in the group. The most informative map was from the TA × KP population. This map was then used in JoinMap4 to provide a starting point for the maps in the other populations which were eventually merged using the map integration functions in JoinMap4 to produce the consensus map.

The resulting TA × KP map contained 600 markers and was used to force the grouping of another population, H × TA. More markers were added to the H × TA groups based on SCL values and identity with other markers. Markers were again removed if they prevented mapping or caused the linkage map to be an unreasonable size, such as 5,000 cM. The TA × KP map was integrated with the resulting H × TA map and this integrated map was used to force grouping in the next population. This procedure was repeated for every population using the newly integrated maps as a starting point for the forced grouping. The order of grouping and population integration into the map was as follows, TA × KP, H × TA, TA Selfs, I × KP, NMBP1243 × KP, Creeper (Cr) × KP, Haden Selfs. After each population was integrated into the map once, TA × KP and H × TA were grouped and integrated for a second time to see if the larger integrated maps could bring in more associated markers and reduce the total length of the maps of each linkage group.

### Trait association

Phenotype data for 14 qualitative traits were available for TA × KP, Cr × KP, and I × KP populations. In all cases KP was the pollen donor as it is polyembryonic. The qualitative traits measured were: stage of fruit ripeness, fruit shape, ground skin color, blush color, blush intensity, bloom, stem end shape, cleavage, beak shape, pulp color, embryo type, flavor, branch habit, tree vigor, beak shape, and cleavage (Table 3). Embryo type was measured by visual inspection of the seed without seed coat from the F_1_mapping population parent (Aron et al., 1998).

**Table 3:** **Fourteen phenotypic traits and their assessment criteria used for trait association in three mapping populations (TA × KP, Cr × KP, and I × KP)**.

**Trait**	**Rating**	**Score description**
Stage of ripeness	0	Hard (no give in fruit)
	1	Rubbery (slight give in fruit under strong thumb pressure)
	2	Sprung (flesh deforms by 2–3 mm with moderate thumb pressure)
	3	Firm soft (whole fruit deforms with moderate hand pressure)
	4	Eating soft (whole fruit deforms with soft hand pressure)
Fruit shape	1	Long
	2	Ovate
	3	Round
Ground skin color	1	Green
	2	Green/yellow
	3	Yellow
	4	Orange
	5	Pink
Blush color	1	Orange
	2	Pink
	3	Red
	4	Burgundy
Blush intensity	1	No blush
	2	Blush barely visible
	3	Slight blush (similar to Kensington Pride)
	4	Medium blush (similar to Haden)
	5	Solid blush (similar to Tommy Atkins)
Bloom (the efflorescence of the wax covering the fruit)	1	Heavy
	2	Light
Stem end shape	1	Deep
	2	Slightly depressed
	3	Level
	4	Slightly raised
	5	Pointed
Pulp color^a^	1	Orange group 24A
	2	Yellow orange group 32A
	3	Yellow group 15A
	4	Yellow group 13B
	5	Yellow group 6A
Embryo type	1	Monoembryonic
	2	Polyembryonic
Flavor	1	Unacceptable
	2	Floridian
	3	Indian
	4	Other
	5	Kensington Pride
	6	South East Asian
Branch habit	1	Upright
	2	Spreading
	3	Intermediate
Tree vigor	1	Extreme dwarf
	2	Dwarf
	3	Low vigor
	4	Medium vigor
	5	High vigor
Beak shape (prominence of the point at the stylar scar)	1	Absent
	2	Very slight
	3	Slight
	4	Medium
	5	Prominent
Cleavage (severity of the groove on the ventral shoulder of the fruit)	1	Deep
	2	Shallow
	3	Absent

a *The_Royal_Horticultural_Society, 2001*.

Of the 14 traits, the twelve fruit traits were assessed on a sample of ten randomly picked at fruit maturity from each individual genotype within the three mapping populations. Fruit were ripened at 26°C and assessed at the eating ripe stage using the criteria detailed in Table 3.

Associating traits with the mapped SNP markers was done using MapQTL6 (Kyazma B.V.®, Wageningen, Netherlands) using Cross Pollinated (CP) for population type and Interval Mapping (IM) for association statistic. All calculation parameters were set to MapQTL6 defaults. Global thresholds were calculated as described in MapQTL6 (permutation tests of 10,000 rounds) and only traits that showed higher association probabilities than the global threshold were considered to be significant.

## Results

### Segregation of SNP markers in the seven mapping populations

Markers were chosen that segregated in a disomic fashion to produce our genetic map. From the 1,054 SNP markers used to genotype the 775 individuals from the seven mapping populations, 56 were removed due to excess missing data, 25 were removed due to aberrant segregation patterns, 19 had two homozygous parents, and 66 were unmappable across all populations for a combination of these reasons such as missing data in one mapping population and aberrant segregation in another, leaving 888 potentially mappable markers (Table 4). As the seven mapping populations had different parents, different sets of markers were mappable within different populations. To merge individual maps into a consensus map required the removal of certain markers that did not appear to be stably inherited in the same position or order in all the mapping populations. In general, these markers were heterozygous in both parents and distant from markers that were heterozygous in only one parent so that correct phasing of the markers in each population was difficult. Thus, although addition of map data from populations with different parents increased the number of markers in the consensus map, it also could lead to the removal of markers that could not be phased correctly. Examples of markers with aberrant segregation patterns for disomic inheritance in different populations are listed in Table 5.

**Table 4:** **Types of markers removed prior to genetic mapping**.

**Type of marker**	**Number of markers**
Total markers	1,054
Aberrant segregation types in all populations	−25
Homozygote × Homozygote in all populations	−19
Too much missing data in all populations	−56
Unmappable in all populations because of a combination of unmappable marker types (e.g., aberrant segregation in one population, missing data in one population, etc.)	−66
Final mappable markers in at least one population	888

**Table 5:** **Examples of aberrant segregation types for SNP markers in a mapping population**.

**Population**	**Marker**	**Segregation ratio (XX:XY:YY:ZZ)**
H × TA	Mi_0299	60:26:63:76
TA × KP	Mi_0020	57:0:43:3
	Mi_0171	50:1:49:3
TA Self pollinated	Contig 1638_A98G	0:66:0:0
	Mi_0103	50:0:16:0
I × KP	Mi_0200	121:3:52:3
NMBP1243 × KP	Mi_0425	72:4:23:1
	Contig 6698_C90T	16:52:32:0

*Markers exhibited aberrant segregation types in one or more populations and were removed from further analysis. Only three genotypes are distinguishable using SNP assays: Homozygous allele 1 (XX), heterozygous (XY), and homozygous allele 2 (YY). If alleles are inherited as in a diploid, expected segregation ratios dependent on possible parental genotypes are: 1:1 XX:XY, 1:2:1 XX:XY:YY, and 1:1 XY:YY. In the aberrant segregation patterns, ZZ represents either a potential null allele or missing data*.

### Consensus genetic map

**Figure 1:**
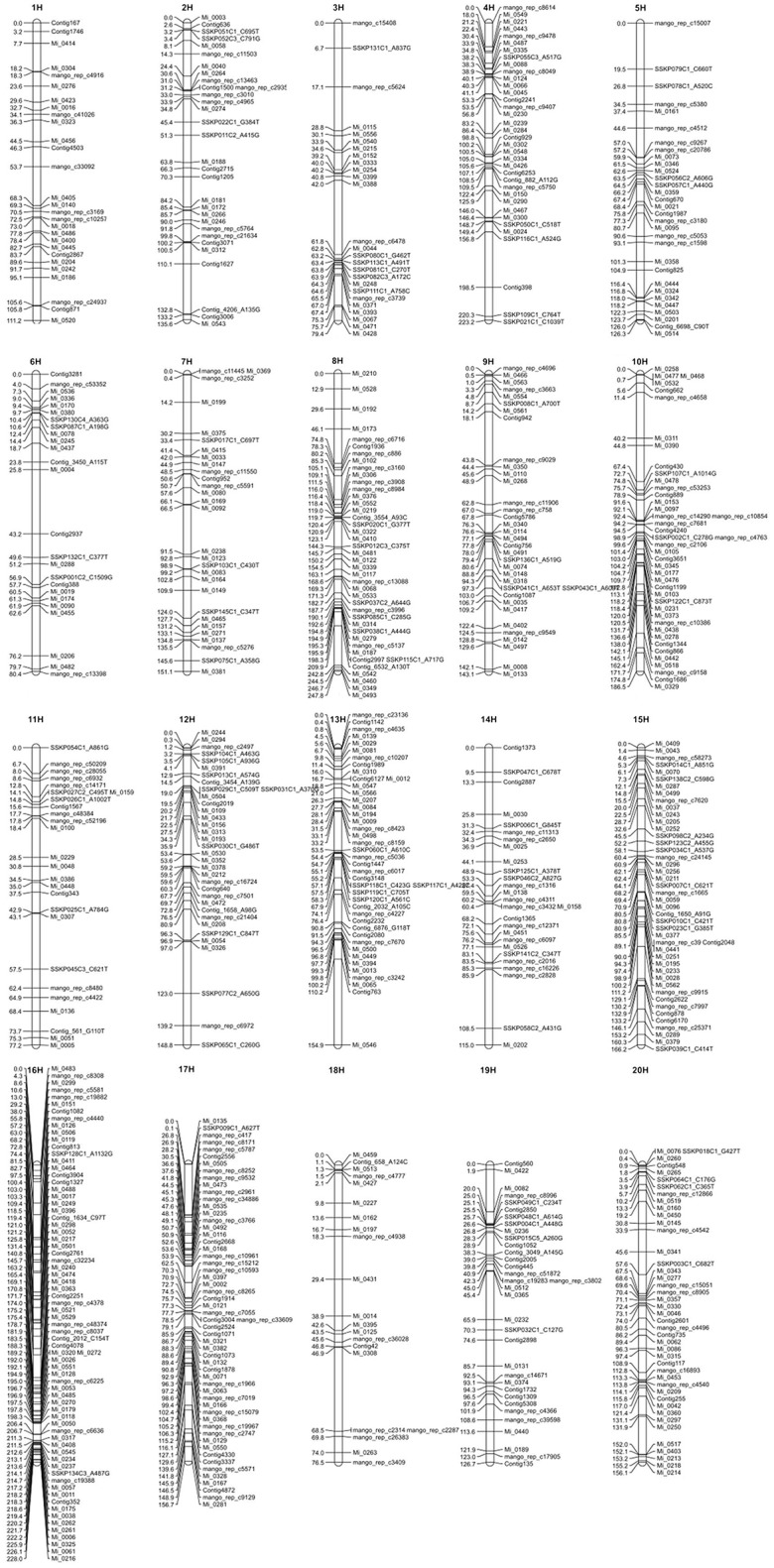
**The consensus genetic map of mango**. Vertical lines represent linkage groups. Horizontal lines crossing the vertical lines depict the name and position in cM of SNP markers on the linkage group.

To include all markers in the consensus map, we employed the strategy detailed in Section Materials and Methods, using the strengths of both JoinMap4 and OneMap. We produced a consensus map with 726 SNP markers distributed across 20 LGs shown in Figure 1. A text version of SNP markers, linkage group and map positions is provided in Table S1. Sequences for the SNP markers, map positions, and annotation, where possible, are presented in Table S2 and Fluidigm assay designs are in Table S3.

Table 6 shows the calculated length in centimorgans (cM) and the number of markers for each of the 20 LGs. Linkage group 8 was the longest at 247.8 cM and LG 16 had the greatest number of markers at 71. Average distance between markers for each LG is also shown in Table 6 and the overall average distance between markers was 4.095 cM. Greatest distance between markers was 44.775 cM on LG 13 and shortest distance was 0.001 cM on LG 8 and 13 not including identical markers (0.000 cM distance). Although, SNP markers had been designed so that there was only one marker per transcript/gene, several SNP markers were mapped to the identical position in all mapping populations suggesting that the 775 meiotic events across all the populations were not sufficient to observe recombination between these genes.

**Table 6:** **Consensus map statistics**.

**LG**	**Number of markers per linkage group**	**Length of each linkage group (cM)**	**Ave distance between markers (cM)**	**Max distance between markers (cM)**	**Min distance between markers (cM)**
1	28	111.2	4.1	14.6	0.06
2	31	135.6	4.5	22.8	0.05
3	26	79.4	3.2	19.8	0.08
4	36	223.2	6.4	41.6	0.07
5	31	126.3	4.2	19.4	0.18
6	25	80.4	3.4	17.4	0.17
7	29	151.1	5.4	25.0	0.00
8	42	247.8	6.0	32.9	0.00
9	35	143.1	4.2	25.7	0.01
10	42	186.5	4.5	28.8	0.00
11	26	77.2	3.1	14.4	0.00
12	35	148.8	4.4	26.1	0.00
13	43	154.9	3.7	44.8	0.00
14	27	114.9	4.4	22.6	0.02
15	45	166.2	3.8	18.0	0.00
16	71	228.0	3.3	17.9	0.00
17	56	156.7	2.8	26.7	0.00
18	21	76.5	3.8	21.6	0.00
19	34	126.7	3.8	20.5	0.00
20	43	156.1	3.7	20.1	0.02
Total	726	2890.6			

*Summary of the final consensus linkage map containing 726 markers across 20 linkage groups. For markers in a linkage group, the minimum number was 21, the maximum number was 71, and the average was 36. For length in cM of a linkage group, the minimum was 76.5 cM, the maximum was 247.8 cM, and the average was 144.5 cM*.

Assuming a haploid genome size of ~439 Mb and 20 chromosomes per haploid genome, the average size of a chromosome would be ~22 Mb. The total size of the map is 2,890 cM. An estimate of the average size of a cM would be ~150 Kb but would be expected to vary greatly within the genome.

### Associating qualitative traits with the map

Qualitative phenotypic data were available for three of the mapping populations (TA × KP, I × KP, and Cr × KP). Interval mapping testing using MapQTL found seven of the 14 qualitative traits used in the association study had significant LOD scores in at least one of the populations. Table 7 shows the seven qualitative traits with significant LOD scores and their position on the map associated with the trait. Reported LOD scores are all above the thresholds determined by permutation tests for the trait in the respective population.

**Table 7:** **Trait association in three mapping populations**.

**Trait**	**LG**	**Marker**	**Position (cM)**	**TA × KP LOD**	**Cr × KP LOD**	**I × KP LOD**
Embryo type	8	Mi_0173	46.1	4.96	8.82	
	8	mango_rep_c6716	74.8		7.70	
	8	Contig1936	78.3		7.40	
	8	mango_rep_c886	80.2		7.23	
	8	Mi_0102	85.3		6.65	
Ground skin color	17	Mi_0135	0.0	5.61		
	17	SSKP009C1_A627T	0.1	5.61		
	20	Mi_0450	19.2			4.62
	20	Mi_0145	30.8			5.83
	20	mango_rep_c4542	33.9			6.17
Blush intensity	20	Mi_0341	45.6			6.65
	20	SSKP003C1_C682T	57.6			5.99
	20	Mi_0343	67.5			5.75
	20	Mi_0277	68.6			5.69
	20	mango_rep_c15051	69.6			5.62
	20	mango_rep_c8905	70.4			5.60
	20	Mi_0357	71.1			5.57
	20	Mi_0330	72.4			5.49
	20	Mi_0046	73.1			5.43
	20	Contig2601	74.0			5.33
Bloom	13	Contig1142	0.4	5.80		
	9	Mi_0417	109.2			4.86
	9	Mi_0402	122.4			8.05
	9	mango_rep_c9549	124.5			7.91
	9	Mi_0142	128.8			7.14
	9	Mi_0497	129.6			7.03
Beak shape	11	mango_c48384	17.7	6.16		
	11	mango_rep_c52196	17.8	6.16		
Pulp color	16	Mi_0217	125.8	5.18		
	13	Mi_0029	5.6			4.36
Branch habit	8	Mi_0192	29.6	4.90		
	16	Contig3904	97.5			4.48
	16	Contig1327	100.4			4.42

*LG, linkage group; TA × KP, Tommy Atkins × Kensington Pride; Cr × KP, Creeper × Kensington Pride; I × KP, Irwin × Kensington Pride; LOD, likelihood of the odds*.

**Figure 2:**
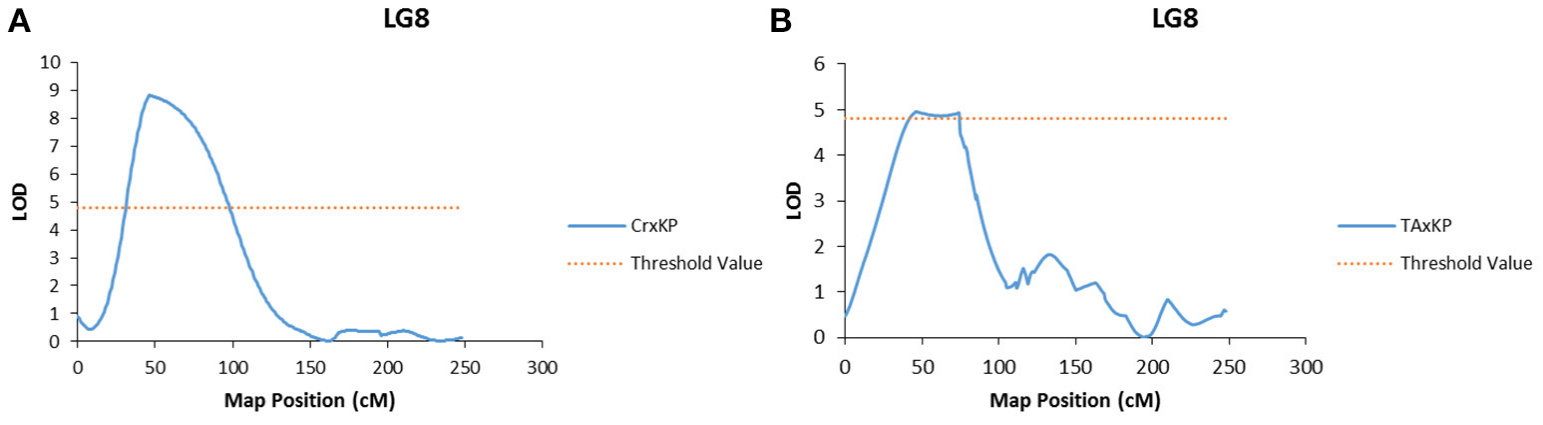
**Graphs of the plot of the likelihood of the odds that a SNP marker is associated with the trait of polyembryony. (A)** Linkage group 8 of the Cr × KP map. **(B)** Linkage group 8 of the TA × KP map.

Embryo type was the only trait to have significant LOD scores at the same marker (Mi_0173) across two different populations (Figure 2). Marker Mi_0173 was unable to be mapped in the I × KP population, which prevented testing for a significant signal for embryo type in that population. For trait association, only genotype data from mapped markers in the population were used to ensure that the phasing specific to the population was correct.

**Figure 3:**
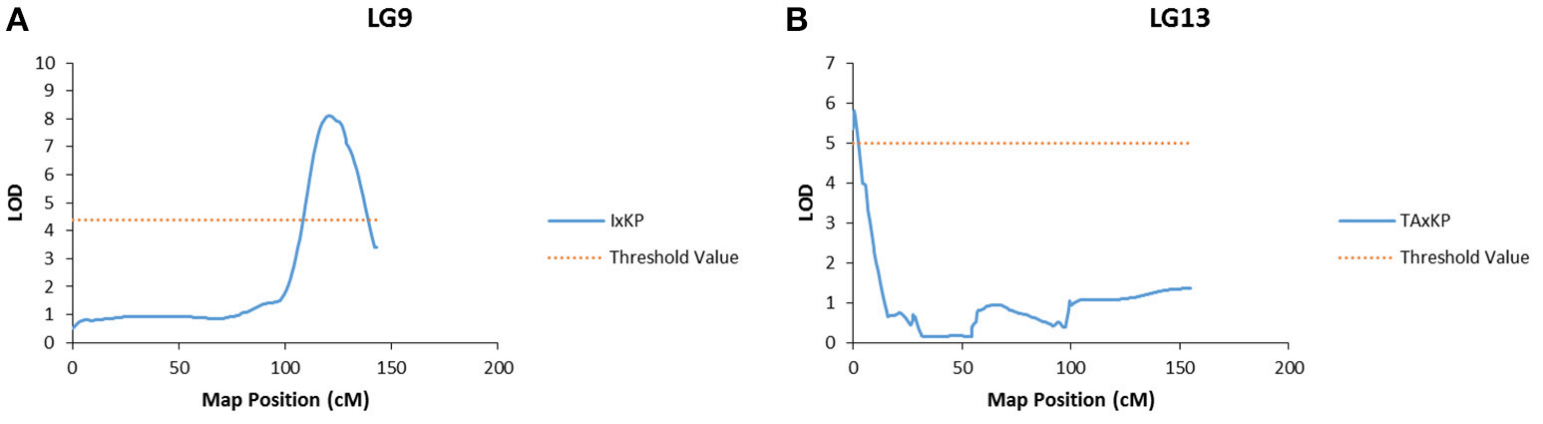
**Graphs of the plot of the likelihood of the odds that a SNP marker is associated with the trait of bloom. (A)** Linkage group 9 of the I × KP map. **(B)** Linkage group 13 of the TA × KP map.

Bloom, pulp color, and branch habit traits showed significant association to markers in two different populations. The marker association was on different LGs in each population (Table 7). For example, the bloom trait showed a significant association to a marker on LG 9 in I × KP and on LG 13 in TA × KP (Figure 3). The ground skin color, blush intensity, and beak shape traits showed a significant association to markers on a single LG in only one population (Table 7).

## Discussion

### A genetic map of mango from SNP markers

MAS provides a means to improve the efficiency of tree breeding. A genetic map provides a means to improve the strength of the association between traits and markers for MAS. We chose to produce a genetic map from SNP markers for several reasons: SNP markers are more abundant than microsatellite markers, easier to identify, easier to score and, as unambiguous markers, are appropriate for international databases as they show no platform bias, which means they can be assayed by any method and produce the same genotype. For mango, ~500,000 SNP markers were identified from RNA sequencing and alignment to a consensus transcriptome (Hoang et al., 2015; Sherman et al., 2015; Kuhn et al., 2016). From these SNPs, 1,054 were selected, converted into assays and used to genotype seven different extant mapping populations of mango comprising 775 individuals. Of the 1,054 SNP markers, 726 segregated in a disomic (Mendelian) fashion, showed normal segregation ratios in at least one of the mapping populations, and could be placed on the genetic map. We also found markers whose segregation could best be explained with a tetrasomic inheritance model, which provides evidence for at least a partial allopolyploid nature of mango. Some markers had aberrant segregation patterns that could not be explained by either a diploid or polyploid model. Not all the markers that showed disomic segregation were able to be assigned to a linkage group. This occurred most frequently when both parents were heterozygous for the marker. In a diploid, when both parents are heterozygous, the phase of the marker must be determined by relating it to the inheritance of the nearest markers where only one parent is heterozygous. In essence, the haplotypes of the parental chromosome pairs are being inferred. However, in a polyploid, there are many more potential combinations of parental haplotypes and, thus, the phase of each haplotype may not be correctly identified. In this situation, the position of the marker on the map may vary dramatically from one population to the next and the marker may also cause significant distortion of the map. In such cases, the marker was removed from the consensus map, unless, in at least one of the mapping populations, only one of the parents was heterozygous for the marker and phase calculation was unnecessary.

Mango has 40 chromosomes with the diploid number being 20. The markers we used for the map were inherited in a disomic fashion, leading to an expectation that we would find 20 identifiable LGs. This suggests that if mango is an allopolyploid, the two ancestral genomes are different enough to be distinguished by our markers.

We used a strategy to make the map that took advantage of the strengths of two different mapping programs, JoinMap4 and OneMap. Using OneMap we set the group size and group number parameters to artificially identify 20 LGs with at least 10 markers per LG. We then used these groups to force group formation using JoinMap4 and to identify a SCL value of markers that were not in the group identified by OneMap. Groups were expanded by setting a minimum SCL value for inclusion into the group and recursively applying this rule until all possible markers with an SCL value over a set threshold had been included in the group. At that point, the larger JoinMap group was used to force group formation in the next mapping population until all possible markers were included in the group. We started this process in OneMap with the TA × KP population as the data for this population showed the least segregation distortion, likely due to the accuracy of the parental genotypes. Using either hand-pollination or open-pollination to create a population of F1 hybrid individuals, the assumption is that all clones of a cultivar that are potential parents have identical genotypes. Thus, there should be no problem with using multiple trees of a cultivar as a parent, rather than a single tree. However, in both hand-pollinated and open-pollinated populations, there may be genotypic differences in the multiple trees used as parents. These slight genotypic differences may not be easily detectable when using a few diagnostic markers, but may be detected when more markers are applied or when segregation distortion in that population for some markers is observed. The TA × KP population had the least amount of this type of distortion, perhaps due to the genetic identity of all the TA and KP clones used as parents. In contrast, the I × KP population, although almost twice as large as the TA × KP population (180:100), had off types identified when all 1,054 markers were used as well as significant distortion that may have been due to the use of several Irwin maternal parents that were not completely identical in genotype. The I × KP map had many fewer mapped markers than the TA × KP map and did not contribute new markers to the consensus map that were unique to I × KP.

### Qualitative trait association to the genetic map

To be useful for MAS, important agronomic traits must be associated with markers. A map is not necessary to identify markers associated with a trait, but confidence in this association increases as multiple markers near the trait locus on the genetic map also show significant association with the trait. This was the case for seven of 14 of our qualitative traits used for the initial trait association studies.

#### Polyembryony

Mango has its origins in Southeast Asia, primarily in the area from north-western Myanmar, Bangladesh, and north-eastern India. From these origins, two centers of diversity developed. A subtropical group in the Indian sub-continent that is characterized by monoembryonic seed and a tropical group in the south-east-Asia region that is characterized by polyembryonic seed (Mukherjee and Litz, 2009).

In our case, embryony type, which is dimorphic (monoembryonic or polyembryonic) showed significant association to a single locus on LG 8 in two of the three mapping populations (Table 7). In crosses between a monoembryonic maternal parent (I, TA, or Cr) and a polyembryonic paternal parent (KP), polyembryony segregated 1:1. One possible explanation for this segregation pattern proposed by Aron et al. (1998) is that the gene regulating polyembryony is heterozygous with a dominant polyembryony allele. Monoembryonic individuals are homozygous recessive. The marker Mi_0173 (LG 8) shows a significant association with the polyembryony trait in both TA × KP and Cr × KP. This is expected as the dominant allele is coming from the same polyembryonic parent (KP). No marker association with polyembryony was seen in I × KP. This may have been due to the inability to map Mi_0173 in the I × KP mapping population as discussed above. In preliminary use of Mi_0173 to screen a germplasm collection, significant association of this marker to the polyembryony trait was also observed (data not shown), suggesting that the position of the trait on LG 8 is not specific to the polyembryonic KP parent common to four of the mapping populations. Our trait association data supports Aron's model of the genetic regulation of polyembryony. The parents of the mapping populations in this study do not adequately represent the genetic diversity of either mono- or polyembryonic cultivars available in germplasm collections. Further dissection of the polyembryony trait in crosses between more genetically diverse cultivars of different origins will increase our understanding of the genetics of this important trait.

#### Other horticultural traits

We saw significant associations of six other traits to specific loci on the genetic map: bloom, pulp color, branch habit, ground skin color, blush intensity, and beak shape. Bloom, pulp color, and branch habit showed association to markers in two different mapping populations (TA × KP, I × KP), but on different linkage groups in each. These traits may be regulated differently in the different accessions. For example, the bloom trait is the amount of wax efflorescence covering the fruit and it was scored as light (I and KP) and heavy (TA). A potential explanation would be that the heavy phenotype for bloom in TA requires activation of wax biosynthetic genes to increase wax production, while the light phenotype in I and KP activates other pathways that use the same long chain fatty acid precursors and reduce wax production. A similar argument can be made for the pulp color and branch habit traits, which also show association to different loci and LGs in different mapping populations.

Significant association of SNP markers with blush intensity, beak shape, and ground skin color was only observed in TA × KP. For blush intensity, the TA and I parents are scored as a 5, KP is an intermediate 3, and Cr is 1. For beak shape, TA, KP, and I are scored as 4 and Cr as 2. One might expect that the blush trait should map in both TA × KP and I × KP and beak shape should map only in Cr × KP. Our results suggest that these traits are regulated in a more complex manner. For ground skin color, the two markers strongly associated with this trait in TA × KP are found at 0 cM and 0.1 cM on LG 17. The next mapped marker is more than 26 cM distant. These two markers only mapped in TA × KP and thus this region of the linkage group cannot be seen in the other populations.

### Allopolyploidy?

We observed segregation patterns of markers that fit more closely to tetrasomic inheritance. For example, in the NMBP1243 × KP population, Mi_0055 showed a segregation pattern of 0:25:75:0 (Homozygous Allele1: Heterozygous: Homozygous Allele2: Missing data or null allele). No parental combination of genotypes for diploid parents could produce such a segregation pattern, but as tetraploid parents, XYYY × YYYY, where X is Allele 1 and Y is Allele 2, the expected segregation would be 0:1:3:0, which fits closely with the observed ratio.

### Using the map for breeding

We have produced a mango consensus genetic map based on individual maps from seven F1 hybrid populations. The individual maps showed strong agreement which makes the consensus map a powerful tool for comparative mapping and the association of markers and alleles to important horticultural traits. Desirable parents can be selected from germplasm collections based on the presence of favorable alleles for the desired trait and used in either hand-pollination crosses or open-pollination of the maternal parent to increase the efficiency of selection of improved material. The trait-associated SNP markers described here can be used to select progeny containing these favorable alleles by genotyping, which is now reliable, rapid, and inexpensive. Genotyping for these traits at the seedling stage will significantly reduce the expense in field use, maintenance and evaluation of material over years. The map opens the way for MAS in mango breeding.

MAS is an excellent tool for preselection of seedlings more likely to show improved traits, but in many fruit tree crops the required genetic resources are not available. The set of markers and genetic map we developed are valuable resources for mango breeders, helping them identify accessions as potential parents and validate progeny as hybrids. The markers and map are a significant step toward improving the efficiency of both traditional breeding and selection through early identification of progeny with trait- and allele-associated genotypes.

The consensus map and qualitative trait-associated markers presented here are the first for mango and demonstrate the utility of such genomics tools for breeding and selection of improved mango cultivars. However, markers associated with important quantitative traits are also needed to further improve mango breeding efficiency. Recently, we have begun a project to produce a map of the TA × KP population by genotyping by sequencing (GBS). The GBS map should be based on more than 100,000 SNP markers and provide the appropriate resolution for the association of quantitative traits to SNP markers for the TA × KP population and, by extension, to other mango hybrid populations with sufficient amounts of accurate phenotypic data.

## Author contributions

DK, IB, ND—mango mapping populations; DK, DI, AS, RO, YC—SNP markers; DK, AG, JR—data reformatting and mapping; DK, IB, ND, DI, AG, JR, RO, YC, AS—conception and design of the work, drafting, and revising the manuscript.

## Funding

DK, AG, JR were funded by USDA-ARS CRIS #6631-21000-022-00D and the National Mango Board NACA#58-6038-5-001. AS, RO were funded by MOAG Chief scientist grant 203-859. YC was funded by MOAG Chief scientist grant 203-088. ND, IB were funded by QDAF, Australia, #HF10189 and Horticulture Innovation Australia (HIA) #MG12015.

### Conflict of interest statement

The authors declare that the research was conducted in the absence of any commercial or financial relationships that could be construed as a potential conflict of interest.

## References

[B1] Aron Y. Czosnek H. Gazit S. Degani C. (1998). Polyembryony in mango (*Mangifera indica* L.) is controlled by a single dominant gene. Hortscience 33, 1241–1242.

[B2] Arumuganathan K. Earle E. D. (1991). Nuclear DNA content of some important plant species. Plant Mol. Biol. Rep. 9, 208–218. 10.1007/BF02672069

[B3] Asker S. Jerling L. (1992). Apomixis in Plants. Boca Raton, FL: CRC press.

[B4] Azim M. K. Khan I. A. Zhang Y. (2014). Characterization of mango (*Mangifera indica* L.) transcriptome and chloroplast genome. Plant Mol. Biol. 85, 193–208. 10.1007/s11103-014-0179-8 24515595

[B5] Bally I. S. E. Lu P. Johnson P. (2009). Mango breeding, in Breeding Plantation Tree Crops: Tropical Species 1st Edn, eds Jain S. M. Priyadarshan P. M. (New York, NY: Springer), 51–82.

[B6] Dautt-Castro M. Ochoa-Leyva A. Contreras-Vergara C. A. Pacheco-Sanchez M. A. Casas-Flores S. Sanchez-Flores A.. (2015). Mango (*Mangifera indica* L.) cv. Kent fruit mesocarp *de novo* transcriptome assembly identifies gene families important for ripening. Front. Plant Sci. 6:62. 10.3389/fpls.2015.00062 25741352 PMC4332321

[B7] Dillon N. L. Bally I. S. E. Wright C. L. Hucks L. Innes D. J. Dietzgen R. G. (2013). Genetic diversity of the australian national mango genebank. Sci. Hortic. 150, 213–226. 10.1016/j.scienta.2012.11.003

[B8] Duval M. F. Bunel J. Sitbon C. Risterucci A. M. (2005). Development of microsatellite markers for mango (*Mangifera indica* L.). Mol. Ecol. Notes 5, 824–826. 10.1111/j.1471-8286.2005.01076.x

[B9] Galán Saúco V. (2013). Worldwide mango production and market: current situation and future prospects. Acta Hortic. 992, 37–48. 10.17660/ActaHortic.2013.992.2

[B10] Harel-Beja R. Sherman A. Rubinstein M. Eshed R. Bar-Ya'akov I. Trainin T. (2015). A novel genetic map of pomegranate based on transcript markers enriched with QTLs for fruit quality traits. Tree Genet. Genomes 11, 1–18. 10.1007/s11295-015-0936-0

[B11] Hoang V. L. Innes D. J. Shaw P. N. Monteith G. R. Gidley M. J. Dietzgen R. G. (2015). Sequence diversity and differential expression of major phenylpropanoid-flavonoid biosynthetic genes among three mango varieties. BMC Genomics 16:561. 10.1186/s12864-015-1784-x 26220670 PMC4518526

[B12] Kashkush K. Jinggui F. Tomer E. Hillel J. Lavi U. (2001). Cultivar identification and genetic map of mango (*Mangifera indica*). Euphytica 122:129 10.1023/A:1012646331258

[B13] Kuhn D. N. Dillon N. L. Innes D. J. Wu L.-S. Mockaitis K. (2016). Development of single nucleotide polymorphism (SNP) markers from the mango (*Mangifera indica*) transcriptome for mapping and estimation of genetic diversity. Acta Hortic. 1111, 315–322. 10.17660/ActaHortic.2016.1111.45

[B14] Kumar V. Malik S. K. Pal D. Srinivasan R. Bhat S. R. (2014). Comparative transcriptome analysis of ovules reveals stress related genes associated with nucellar polyembryony in citrus. Tree Genet. Genomes 10, 449–464. 10.1007/s11295-013-0690-0

[B15] Litz R. E. (2009). The Mango: Botany, Production and Uses. Wallingford: CABI.

[B16] Luo C. Shu B. Yao Q. Wu H. Xu W. Wang S. (2016). Construction of a high-density genetic map based on large-scale marker development in mango using specific-locus amplified fragment sequencing (SLAF-seq). Front. Plant Sci. 7:1310. 10.3389/fpls.2016.01310 27625670 PMC5003885

[B17] Luria N. Sela N. Yaari M. Feygenberg O. Kobiler I. Lers A.. (2014). *De-novo* assembly of mango fruit peel transcriptome reveals mechanisms of mango response to hot water treatment. BMC Genomics 15:957. 10.1186/1471-2164-15-957 25373421 PMC4236434

[B18] Margarido G. R. Souza A. P. Garcia A. A. (2007). OneMap: software for genetic mapping in outcrossing species. Hereditas 144, 78–79. 10.1111/j.2007.0018-0661.02000.x 17663699

[B19] Martínez-García P. J. Parfitt D. E. Ogundiwin E. A. Fass J. Chan H. M. Ahmad R. (2013). High density SNP mapping and QTL analysis for fruit quality characteristics in peach (*Prunus persica* L.). Tree Genet. Genomes 9, 19–36. 10.1007/s11295-012-0522-7

[B20] Mukherjee S. K. (1950). Mango: its allopolyploid nature. Nature 166, 196–197. 10.1038/166196b0 15439231

[B21] Mukherjee S. K. Litz R. E. (2009). Introduction: botany and importance, in The Mango; Botany, Production and Uses 2nd Edn, ed Litz R. E. (Wallingford, CT; Oxen: CAB International), 1–18.

[B22] Nakano M. Kigoshi K. Shimizu T. Endo T. Shimada T. Fujii H. (2013). Characterization of genes associated with polyembryony and *in vitro* somatic embryogenesis in Citrus. Tree Genet. Genomes 9, 795–803. 10.1007/s11295-013-0598-8

[B23] Nakano M. Shimada T. Endo T. Fujii H. Nesumi H. Kita M.. (2012). Characterization of genomic sequence showing strong association with polyembryony among diverse Citrus species and cultivars, and its synteny with Vitis and Populus. Plant Sci. 183, 131–142. 10.1016/j.plantsci.2011.08.002 22195586

[B24] Ogundiwin E. A. Peace C. P. Gradziel T. M. Parfitt D. E. Bliss F. A. Crisosto C. H. (2009). A fruit quality gene map of Prunus. BMC Genomics 10:587. 10.1186/1471-2164-10-587 19995417 PMC2797820

[B25] Pandit S. S. Kulkarni R. S. Giri A. P. Kollner T. G. Degenhardt J. Gershenzon J.. (2010). Expression profiling of various genes during the fruit development and ripening of mango. Plant Physiol. Biochem. 48, 426–433. 10.1016/j.plaphy.2010.02.012 20363641

[B26] Schnell R. J. Brown J. S. Olano C. T. Meerow A. W. Campbell R. J. Kuhn D. N. (2006). Mango genetic diversity analysis and pedigree inferences for Florida cultivars using microsatellite markers. J. Am. Soc. Hortic. Sci. 131, 214–224.

[B27] Schnell R. J. Olano C. T. Quintanilla W. E. Meerow A. W. (2005). Isolation and characterization of 15 microsatellite loci from mango (*Mangifera indica* L.) and cross-species amplification in closely related taxa. Mol. Ecol. Notes 5, 625–627. 10.1111/j.1471-8286.2005.01018.x

[B28] Sherman A. Rubinstein M. Eshed R. Benita M. Ish-Shalom M. Sharabi-Schwager M.. (2015). Mango (*Mangifera indica* L.) germplasm diversity based on single nucleotide polymorphisms derived from the transcriptome. BMC Plant Biol. 15:277. 10.1186/s12870-015-0663-6 26573148 PMC4647706

[B29] Sturrock T. T. (1968). Genetics of mango polyembryony. Fla. State Hort. Soc. Proc. 81, 311–314.

[B30] The_Royal_Horticultural_Society (2001). RHS Colour Chart, 4th Edn. London: Royal Horticultural Society.

[B31] Viruel M. A. Escribano P. Barbieri M. Ferri M. Hormaza J. I. (2005). Fingerprinting, embryo type and geographic differentiation in mango (*Mangifera indica* L., Anacardiaceae) with microsatellites. Mol. Breed. 15, 383–393. 10.1007/s11032-004-7982-x

[B32] Wu H.-X. Jia H.-M. Ma X.-W. Wang S.-B. Yao Q.-S. Xu W.-T.. (2014). Transcriptome and proteomic analysis of mango (*Mangifera indica* Linn) fruits. J. Proteomics 105, 19–30. 10.1016/j.jprot.2014.03.030 24704857

