# Supplementary material for: Genetic Map of Mango: A Tool for Mango Breeding
Source: Front Plant Sci. 2017 Apr 20;8:577. doi: 10.3389/fpls.2017.00577 (PMC5397511; doi:10.3389/fpls.2017.00577)
Supplement: Supplementary file 1 [file Table1.DOCX]

Table S1

SNP Marker Linkage Group Position (cM)

Contig167 1 0

Contig1746 1 3.237

Mi_0414 1 7.743

Mi_0304 1 18.203

mango_rep_c4916 1 18.261

Mi_0276 1 23.612

Mi_0423 1 29.575

Mi_0016 1 32.737

mango_c41026 1 34.127

Mi_0323 1 36.315

Mi_0456 1 44.466

Contig4503 1 46.313

mango_c33092 1 53.673

Mi_0405 1 68.302

Mi_0140 1 69.331

mango_rep_c3169 1 70.451

mango_rep_c10257 1 72.515

Mi_0018 1 72.999

Mi_0486 1 77.819

Mi_0400 1 78.35

Mi_0445 1 82.659

Contig2867 1 83.745

Mi_0204 1 89.59

Mi_0242 1 91.707

Mi_0186 1 95.072

mango_rep_c24937 1 105.604

Contig871 1 105.849

Mi_0520 1 111.24

Mi_0003 2 0

Contig636 2 2.598

SSKP051C1_C695T 2 3.207

SSKP052C3_C791G 2 3.376

Mi_0058 2 8.052

mango_rep_c11503 2 14.322

Mi_0040 2 24.366

Mi_0264 2 30.563

mango_rep_c13463 2 31.009

Contig1500 2 31.195

mango_rep_c2935 2 31.248

mango_rep_c3010 2 32.955

mango_rep_c4965 2 33.92

Mi_0274 2 34.815

SSKP022C1_G384T 2 45.449

SSKP011C2_A415G 2 51.27

Mi_0188 2 63.782

Contig2715 2 66.315

Contig1205 2 70.337

Mi_0181 2 84.19

Mi_0172 2 85.435

Mi_0266 2 85.667

Mi_0246 2 89.951

mango_rep_c5764 2 91.83

mango_rep_c21634 2 99.824

Contig3071 2 100.156

Mi_0312 2 100.517

Contig1627 2 110.064

Contig_4206_A135G 2 132.843

Contig3006 2 133.16

Mi_0543 2 135.589

mango_c15408 3 0

SSKP131C1_A837G 3 6.743

mango_rep_c5624 3 17.139

Mi_0115 3 28.765

Mi_0556 3 30.119

Mi_0540 3 33.941

Mi_0215 3 34.633

Mi_0152 3 39.23

Mi_0333 3 40.03

Mi_0254 3 40.2

Mi_0399 3 40.782

Mi_0388 3 42.022

mango_rep_c6478 3 61.782

Mi_0044 3 62.822

SSKP080C1_G462T 3 63.175

SSKP113C1_A491T 3 63.391

SSKP081C1_C270T 3 63.823

SSKP082C3_A172C 3 63.904

Mi_0248 3 64.33

SSKP111C1_A758C 3 64.644

mango_rep_c3739 3 65.536

Mi_0371 3 66.966

Mi_0393 3 67.393

Mi_0067 3 75.281

Mi_0471 3 75.749

Mi_0428 3 79.433

mango_rep_c8614 4 0

Mi_0549 4 17.974

Mi_0221 4 21.15

Mi_0443 4 22.413

mango_rep_c9478 4 30.377

Mi_0487 4 33.893

Mi_0335 4 34.772

SSKP055C3_A517G 4 38.22

Mi_0088 4 38.293

mango_rep_c8049 4 38.933

Mi_0124 4 40.062

Mi_0066 4 40.346

Mi_0045 4 41.097

Contig2241 4 53.266

mango_rep_c9407 4 53.502

Mi_0230 4 56.822

Mi_0239 4 83.224

Mi_0284 4 86.434

Contig929 4 98.816

Mi_0302 4 100.203

Mi_0548 4 100.469

Mi_0334 4 104.965

Mi_0426 4 105.574

Contig6253 4 107.126

Contig_882_A112G 4 108.477

mango_rep_c5750 4 109.471

Mi_0150 4 122.375

Mi_0290 4 125.903

Mi_0467 4 146.033

Mi_0300 4 146.369

SSKP050C1_C518T 4 148.744

Mi_0024 4 149.379

SSKP116C1_A524G 4 156.84

Contig398 4 198.474

SSKP109C1_C764T 4 220.276

SSKP021C1_C1039T 4 223.198

mango_rep_c15007 5 0

SSKP079C1_C660T 5 19.45

SSKP078C1_A520C 5 26.761

mango_rep_c5380 5 34.521

Mi_0161 5 37.422

mango_rep_c4512 5 44.591

mango_rep_c9267 5 56.983

mango_rep_c20786 5 57.227

Mi_0073 5 59.925

Mi_0346 5 61.485

Mi_0524 5 62.612

SSKP056C2_A606G 5 63.466

SSKP057C1_A440G 5 64.5

Mi_0359 5 66.224

Contig670 5 67.439

Mi_0021 5 68.383

Contig1987 5 75.832

mango_rep_c3180 5 77.269

Mi_0095 5 80.715

mango_rep_c5053 5 90.607

mango_rep_c1598 5 93.107

Mi_0358 5 101.291

Contig825 5 104.867

Mi_0444 5 116.352

Mi_0324 5 116.768

Mi_0342 5 118.015

Mi_0447 5 118.2

Mi_0503 5 122.263

Mi_0201 5 123.73

Contig_6698_C90T 5 125.994

Mi_0514 5 126.276

Contig3281 6 0

mango_rep_c53352 6 3.955

Mi_0536 6 7.294

Mi_0336 6 9.01

Mi_0170 6 9.446

Mi_0380 6 9.692

SSKP130C4_A363G 6 10.404

SSKP087C1_A198G 6 10.57

Mi_0078 6 12.409

Mi_0245 6 14.423

Mi_0437 6 18.657

Contig_3450_A115T 6 23.833

Mi_0004 6 25.765

Contig2937 6 43.205

SSKP132C1_C377T 6 49.648

Mi_0288 6 51.157

SSKP001C2_C1509G 6 56.87

Contig388 6 57.677

Mi_0019 6 60.47

Mi_0174 6 61.327

Mi_0090 6 61.904

Mi_0455 6 62.562

Mi_0206 6 76.218

Mi_0482 6 79.711

mango_rep_c13398 6 80.435

mango_c11445 7 0

Mi_0369 7 0.002

mango_rep_c3252 7 0.449

Mi_0199 7 14.183

Mi_0375 7 30.212

SSKP017C1_C697T 7 33.398

Mi_0415 7 41.446

Mi_0033 7 41.989

Mi_0147 7 44.887

mango_rep_c11550 7 48.485

Contig952 7 50.606

mango_rep_c5591 7 50.741

Mi_0080 7 57.598

Mi_0169 7 66.12

Mi_0092 7 66.52

Mi_0238 7 91.517

Mi_0123 7 92.823

SSKP103C1_C430T 7 98.894

Mi_0083 7 99.198

Mi_0164 7 102.782

Mi_0149 7 109.914

SSKP145C1_C347T 7 124.003

Mi_0465 7 127.738

Mi_0157 7 131.194

Mi_0271 7 133.1

Mi_0137 7 134.773

mango_rep_c5276 7 135.496

SSKP075C1_A358G 7 145.614

Mi_0381 7 151.12

Mi_0210 8 0

Mi_0528 8 12.854

Mi_0192 8 29.62

Mi_0173 8 46.139

mango_rep_c6716 8 74.814

Contig1936 8 78.339

mango_rep_c886 8 80.205

Mi_0102 8 85.347

mango_rep_c3160 8 105.109

Mi_0306 8 109.08

mango_rep_c3908 8 111.508

mango_rep_c8984 8 115.955

Mi_0376 8 116.405

Mi_0552 8 118.415

Mi_0219 8 118.983

Contig_3554_A93C 8 119.722

SSKP020C1_G377T 8 120.41

Mi_0322 8 120.932

Mi_0410 8 123.052

SSKP012C3_C375T 8 144.307

Mi_0481 8 145.674

Mi_0122 8 150.189

Mi_0339 8 154.529

Mi_0117 8 163.122

mango_rep_c13088 8 168.57

Mi_0068 8 169.277

Mi_0533 8 171.349

SSKP037C2_A644G 8 182.744

mango_rep_c3996 8 187.743

SSKP085C1_C285G 8 190.122

Mi_0314 8 192.55

SSKP038C1_A444G 8 194.846

Mi_0279 8 194.885

mango_rep_c5137 8 195.33

Mi_0187 8 195.856

Contig2997 8 198.314

SSKP115C1_A717G 8 198.315

Contig_6532_A130T 8 209.899

Mi_0542 8 242.811

Mi_0460 8 244.508

Mi_0349 8 246.721

Mi_0493 8 247.848

mango_rep_c4696 9 0

Mi_0466 9 0.548

Mi_0563 9 1.047

mango_rep_c3663 9 3.305

Mi_0554 9 4.824

SSKP008C1_A700T 9 8.698

Mi_0561 9 14.196

Contig942 9 18.067

mango_rep_c9029 9 43.776

Mi_0350 9 44.378

Mi_0110 9 45.592

Mi_0268 9 48.946

mango_rep_c11906 9 62.803

mango_rep_c758 9 66.98

Contig5786 9 67.754

Mi_0340 9 76.257

Mi_0114 9 76.612

Mi_0494 9 77.148

Contig756 9 77.818

Mi_0491 9 77.99

SSKP136C1_A519G 9 79.445

Mi_0074 9 80.626

Mi_0148 9 88.849

Mi_0318 9 94.321

SSKP041C1_A653T 9 97.305

SSKP043C1_A609T 9 97.317

Contig1087 9 102.962

Mi_0035 9 106.725

Mi_0417 9 109.215

Mi_0402 9 122.355

mango_rep_c9549 9 124.504

Mi_0142 9 128.837

Mi_0497 9 129.648

Mi_0008 9 142.072

Mi_0133 9 143.132

Mi_0258 10 0

Mi_0477 10 0.713

Mi_0468 10 0.714

Mi_0532 10 0.733

Contig662 10 5.625

mango_rep_c4658 10 11.35

Mi_0311 10 40.166

Mi_0390 10 44.794

Contig430 10 67.439

SSKP107C1_A1014G 10 72.669

Mi_0478 10 74.803

mango_rep_c53253 10 75.738

Contig889 10 78.918

Mi_0153 10 91.619

Mi_0097 10 92.11

mango_rep_c14290 10 92.375

mango_rep_c10854 10 92.432

mango_rep_c7681 10 94.229

Contig4240 10 94.5

SSKP002C1_C278G 10 98.877

mango_rep_c4763 10 98.897

mango_rep_c2106 10 99.57

Mi_0105 10 101.44

Contig3651 10 103.006

Mi_0345 10 104.187

Mi_0177 10 104.692

Mi_0476 10 109.715

Contig1199 10 112.813

Mi_0103 10 113.089

SSKP122C1_C873T 10 118.233

Mi_0231 10 118.387

Mi_0373 10 120.042

mango_rep_c10386 10 120.517

Mi_0438 10 131.666

Mi_0278 10 136.577

Contig1344 10 137.977

Contig866 10 142.09

Mi_0442 10 145.071

Mi_0518 10 162.389

mango_rep_c9158 10 171.735

Contig1686 10 174.79

Mi_0329 10 186.529

SSKP054C1_A861G 11 0

mango_rep_c50209 11 6.714

mango_rep_c28055 11 8.041

mango_rep_c6932 11 8.642

mango_rep_c14171 11 12.777

SSKP027C2_C495T 11 14.13

Mi_0159 11 14.132

SSKP026C1_A1002T 11 14.817

Contig1567 11 15.615

mango_c48384 11 17.685

mango_rep_c52196 11 17.758

Mi_0100 11 18.373

Mi_0229 11 28.535

Mi_0048 11 30.817

Mi_0386 11 34.545

Mi_0448 11 35.024

Contig343 11 37.477

SSKP025C1_A784G 11 42.873

Mi_0307 11 43.05

SSKP045C3_C621T 11 57.464

mango_rep_c8480 11 62.363

mango_rep_c4422 11 64.899

Mi_0136 11 68.44

Contig_561_G110T 11 73.707

Mi_0051 11 75.261

Mi_0005 11 77.238

Mi_0244 12 0

Mi_0294 12 0.345

mango_rep_c2497 12 1.225

SSKP104C1_A463G 12 3.245

SSKP105C1_A936G 12 3.469

Mi_0391 12 4.144

SSKP013C1_A574G 12 12.868

Contig_3454_A139G 12 14.483

SSKP029C1_C509T 12 18.993

SSKP031C1_A370G 12 18.993

Mi_0504 12 19.019

Contig2019 12 19.499

Mi_0109 12 20.238

Mi_0433 12 21.65

Mi_0156 12 22.456

Mi_0313 12 27.541

Mi_0193 12 34.295

SSKP030C1_G486T 12 35.899

Mi_0530 12 53.364

Mi_0352 12 53.607

Mi_0378 12 59.218

Mi_0212 12 59.507

mango_rep_c16724 12 59.618

Contig640 12 60.323

mango_rep_c7501 12 67.721

Mi_0472 12 69.746

Contig_1658_A98G 12 72.772

mango_rep_c21404 12 76.486

Mi_0208 12 80.915

SSKP129C1_C847T 12 96.309

Mi_0054 12 96.948

Mi_0326 12 96.956

SSKP077C2_A650G 12 123.026

mango_rep_c6972 12 139.219

SSKP065C1_C260G 12 148.84

mango_rep_c23136 13 0

Contig1142 13 0.383

mango_rep_c4635 13 0.791

Mi_0139 13 4.473

Mi_0029 13 5.61

Mi_0081 13 6.673

mango_rep_c10207 13 9.754

Contig1989 13 11.407

Mi_0310 13 16.006

Contig6127 13 16.661

Mi_0012 13 16.737

Mi_0547 13 18.767

Mi_0566 13 21.002

Mi_0207 13 26.271

Mi_0084 13 27.711

Mi_0194 13 28.056

Mi_0009 13 28.383

mango_rep_c8423 13 31.473

Mi_0498 13 33.131

mango_rep_c8159 13 33.206

SSKP060C1_A610C 13 53.486

mango_rep_c5036 13 54.414

Contig1447 13 54.683

mango_rep_c6017 13 55.134

Contig3148 13 55.223

SSKP118C1_C423G 13 57.13

SSKP117C1_A422C 13 57.131

SSKP119C1_C705T 13 57.533

SSKP120C1_A561C 13 58.339

Contig_2032_A105C 13 67.926

mango_rep_c4227 13 74.057

Contig2232 13 76.441

Contig_6876_G118T 13 90.816

Contig2080 13 91.496

mango_rep_c7670 13 94.257

Mi_0500 13 96.469

Mi_0449 13 96.781

Mi_0394 13 97.721

Mi_0013 13 99.259

mango_rep_c3242 13 99.776

Mi_0065 13 100.182

Contig763 13 110.173

Mi_0546 13 154.948

Contig1373 14 0

SSKP047C1_C678T 14 9.508

Contig2887 14 13.297

Mi_0030 14 25.776

SSKP006C1_G845T 14 31.325

mango_rep_c11313 14 32.415

mango_rep_c2650 14 34.257

Mi_0025 14 36.869

Mi_0253 14 44.133

SSKP125C1_A378T 14 48.878

SSKP046C2_A827G 14 53.305

mango_rep_c1316 14 57.35

Mi_0138 14 59.468

mango_rep_c4311 14 60.199

mango_rep_c3432 14 60.4

Mi_0158 14 60.42

Contig1365 14 68.215

mango_rep_c12371 14 72.084

Mi_0451 14 75.587

mango_rep_c6097 14 76.191

Mi_0526 14 77.125

SSKP141C2_C347T 14 83.093

mango_rep_c2016 14 83.528

mango_rep_c16226 14 85.25

mango_rep_c2828 14 85.895

SSKP058C2_A431G 14 108.488

Mi_0202 14 114.959

Mi_0409 15 0

Mi_0043 15 1.445

mango_rep_c58273 15 4.604

SSKP014C1_A851G 15 5.299

Mi_0070 15 6.069

SSKP138C2_C598G 15 7.297

Mi_0287 15 12.13

Mi_0499 15 14.836

mango_rep_c7620 15 15.545

Mi_0037 15 20.036

Mi_0243 15 22.531

Mi_0205 15 28.661

Mi_0252 15 32.62

SSKP098C2_A234G 15 45.534

SSKP123C2_A455G 15 52.249

SSKP034C1_A537G 15 58.138

mango_rep_c24145 15 60.435

Mi_0296 15 60.898

Mi_0256 15 62.051

Mi_0211 15 62.374

SSKP007C1_C621T 15 64.13

mango_rep_c1665 15 68.166

Mi_0059 15 69.379

Mi_0096 15 70.884

Contig_1650_A91G 15 80.456

SSKP010C1_C421T 15 80.772

SSKP023C1_G385T 15 80.948

Mi_0377 15 85.504

mango_rep_c39 15 89.142

Contig2048 15 89.142

Mi_0441 15 89.142

Mi_0251 15 89.985

Mi_0195 15 94.284

Mi_0233 15 97.352

Mi_0028 15 98.91

Mi_0562 15 100.215

mango_rep_c9915 15 111.156

Contig2622 15 129.147

mango_rep_c7997 15 130.169

Contig878 15 132.942

Contig6170 15 133.211

mango_rep_c25371 15 146.138

Mi_0289 15 153.197

Mi_0379 15 160.318

SSKP039C1_C414T 15 166.222

Mi_0483 16 0

mango_rep_c8308 16 4.251

Mi_0299 16 8.636

mango_rep_c5581 16 10.559

mango_rep_c19882 16 12.995

Mi_0151 16 29.192

Contig1082 16 37.989

mango_rep_c4440 16 55.843

Mi_0126 16 57.2

Mi_0506 16 63.027

Mi_0119 16 68.179

Contig813 16 72.837

SSKP128C1_A1132G 16 74.409

Mi_0411 16 81.464

Mi_0464 16 82.742

Contig3904 16 97.495

Contig1327 16 100.399

Mi_0488 16 103.022

Mi_0017 16 103.319

Mi_0249 16 109.39

Mi_0396 16 118.476

Contig_1634_C97T 16 119.432

Mi_0298 16 120.973

Mi_0052 16 121.23

Mi_0217 16 125.78

Mi_0501 16 131.355

Contig2761 16 140.777

mango_c32234 16 145.703

Mi_0240 16 163.181

Mi_0474 16 165.392

Mi_0418 16 169.138

Mi_0363 16 170.846

Contig2251 16 171.717

mango_rep_c4378 16 173.972

Mi_0521 16 175.167

Mi_0529 16 175.446

mango_rep_c48374 16 178.703

mango_rep_c8037 16 181.885

Contig_2012_C154T 16 183.521

Contig4078 16 188.329

Mi_0320 16 189.228

Mi_0272 16 189.228

Mi_0026 16 192.014

Mi_0551 16 192.066

Mi_0128 16 194.877

mango_rep_c6225 16 195.011

Mi_0053 16 196.679

Mi_0485 16 196.861

Mi_0270 16 197.467

Mi_0179 16 197.844

Mi_0118 16 198.257

Mi_0050 16 206.363

mango_rep_c6636 16 206.676

Mi_0317 16 211.345

Mi_0408 16 211.548

Mi_0545 16 212.636

Mi_0234 16 213.14

Mi_0237 16 213.551

SSKP134C3_A487G 16 214.07

mango_c19388 16 214.656

Mi_0057 16 217.182

Mi_0011 16 218.179

Contig352 16 218.289

Mi_0175 16 218.559

Mi_0038 16 219.437

Mi_0262 16 220.162

Mi_0261 16 221.743

Mi_0006 16 222.185

Mi_0325 16 225.864

Mi_0061 16 226.079

Mi_0216 16 228.013

Mi_0135 17 0

SSKP009C1_A627T 17 0.14

mango_rep_c417 17 26.828

mango_rep_c8171 17 26.9

mango_rep_c5787 17 28.211

Contig2556 17 30.53

Mi_0505 17 36.553

mango_rep_c8252 17 37.626

mango_rep_c9532 17 41.829

Mi_0473 17 44.519

mango_rep_c2961 17 45.149

mango_rep_c34886 17 45.337

Mi_0535 17 47.558

Mi_0235 17 48.084

mango_rep_c3766 17 49.088

Mi_0492 17 50.687

Mi_0116 17 50.895

Contig2668 17 52.648

Mi_0168 17 53.566

mango_rep_c10961 17 53.906

mango_rep_c15212 17 62.471

mango_rep_c10593 17 70.341

Mi_0397 17 70.859

Mi_0002 17 72.674

mango_rep_c8265 17 74.484

Contig1914 17 75.712

Mi_0121 17 77.307

mango_rep_c7055 17 77.676

Contig3004 17 78.464

mango_rep_c33609 17 78.464

Contig2524 17 79.132

Contig1071 17 85.917

Mi_0321 17 86.684

Mi_0382 17 88.287

Contig1073 17 88.592

Mi_0132 17 89.408

Contig1878 17 90.765

Mi_0071 17 92.919

mango_rep_c1966 17 96.287

Mi_0063 17 97.241

mango_rep_c7019 17 98.619

Mi_0166 17 99.444

mango_rep_c15079 17 102.429

Mi_0368 17 104.689

mango_rep_c19967 17 105.194

mango_rep_c2747 17 106.321

Mi_0129 17 115.184

Mi_0550 17 116.059

Contig4330 17 127.052

Contig3337 17 129.61

mango_rep_c5571 17 139.554

Mi_0328 17 141.794

Mi_0167 17 145.906

Contig4872 17 146.49

mango_rep_c9129 17 148.908

Mi_0281 17 156.703

Mi_0459 18 0

Contig_658_A124C 18 1.126

Mi_0513 18 1.347

mango_rep_c4777 18 1.487

Mi_0427 18 2.14

Mi_0227 18 9.798

Mi_0162 18 13.56

Mi_0197 18 16.683

mango_rep_c4938 18 18.319

Mi_0431 18 29.351

Mi_0014 18 38.933

Mi_0395 18 42.562

Mi_0125 18 43.451

mango_rep_c36028 18 45.588

Contig42 18 46.805

Mi_0308 18 46.885

mango_rep_c2314 18 68.45

mango_rep_c2287 18 68.452

mango_rep_c26383 18 69.833

Mi_0263 18 74.017

mango_rep_c3409 18 76.515

Contig560 19 0

Mi_0422 19 1.86

Mi_0082 19 20.042

mango_rep_c8996 19 25.018

SSKP049C1_C234T 19 25.144

Contig2850 19 25.506

SSKP048C1_A614G 19 25.689

SSKP004C1_A448G 19 26.592

Mi_0236 19 26.846

SSKP015C5_A260G 19 28.294

Contig1052 19 28.913

Contig_3049_A145G 19 38.296

Contig2005 19 39.028

Contig445 19 39.75

mango_rep_c51872 19 40.919

mango_c19283 19 42.272

mango_rep_c3802 19 42.272

Mi_0512 19 44.988

Mi_0365 19 45.432

Mi_0232 19 65.904

SSKP032C1_C127G 19 70.309

Contig2898 19 74.586

Mi_0131 19 85.663

mango_c14671 19 92.501

Mi_0374 19 93.1

Contig1732 19 94.289

Contig1309 19 96.505

Contig5308 19 97.573

mango_rep_c4366 19 101.912

mango_rep_c39598 19 108.562

Mi_0440 19 113.604

Mi_0189 19 121.896

mango_rep_c17905 19 122.994

Contig135 19 126.69

Mi_0076 20 0

SSKP018C1_G427T 20 0.023

Mi_0260 20 0.389

Contig548 20 0.891

Mi_0265 20 1.76

SSKP064C1_C176G 20 3.469

SSKP062C1_C365T 20 3.944

mango_rep_c12866 20 5.659

Mi_0519 20 10.2

Mi_0160 20 13.255

Mi_0450 20 19.22

Mi_0145 20 30.756

mango_rep_c4542 20 33.939

Mi_0341 20 45.595

SSKP003C1_C682T 20 57.583

Mi_0343 20 67.468

Mi_0277 20 68.592

mango_rep_c15051 20 69.614

mango_rep_c8905 20 70.406

Mi_0357 20 71.12

Mi_0330 20 72.411

Mi_0046 20 73.076

Contig2601 20 74.009

mango_rep_c4496 20 80.525

Contig735 20 86.225

Mi_0062 20 89.366

Mi_0086 20 96.316

Mi_0315 20 97.411

Contig117 20 108.923

mango_c16893 20 112.809

Mi_0453 20 113.267

mango_rep_c4540 20 113.765

Mi_0209 20 114.142

Contig255 20 115.822

Mi_0042 20 116.996

Mi_0360 20 121.434

Mi_0297 20 131.107

Mi_0250 20 131.869

Mi_0517 20 151.993

Mi_0403 20 152.078

Mi_0213 20 153.201

Mi_0218 20 155.214

Mi_0214 20 156.067
